# Sustainable development through eco-innovation: A focus on small and medium enterprises in Colombia

**DOI:** 10.1371/journal.pone.0316620

**Published:** 2025-01-16

**Authors:** Thomas Tegethoff, Ricardo Santa, Juan Manuel Bucheli, Benjamin Cabrera, Annibal Scavarda

**Affiliations:** 1 Colegio de Estudios Superiores de Administración–CESA, Bogotá, Colombia; 2 Institución Universitaria Escuela Nacional del Deporte, Cali, Colombia; 3 Universidad Icesi, Cali, Colombia; 4 Federal University of the State of Rio de Janeiro, Rio de Janeiro, Brazil; West Pomeranian University of Technology, POLAND

## Abstract

This study examines the impact of eco-innovation on the economic, social, and environmental performance of small and medium enterprises (SMEs) in Colombia. SMEs are pivotal to Colombia’s economic landscape, contributing significantly to job creation, economic growth, and regional development. The research utilizes structural equation modeling (SEM) to analyze data collected from 568 SMEs through an electronic survey. The findings indicate that eco-innovation positively influences both environmental and economic-social performance. Enhanced environmental performance, driven by eco-innovation, is associated with improved resource efficiency, reduced emissions, and waste management. Moreover, economic and social performance, measured through profitability, product quality, and job satisfaction, also benefits from eco-innovative practices. These results underscore the importance of eco-innovation in promoting sustainable development within the SME sector. The study advocates for further large-scale investigations to validate these findings and to explore the broader implications of eco-innovation in diverse economic contexts.

## Introduction

Environmental economics has undergone a fundamental transformation in recent decades, signaling a shift towards a more holistic understanding of environmental issues. Valencia et al. [1] emphasize that this evolution entails a growing recognition of the importance of valuing ecosystem services in decision-making, reflecting a shift towards considering use values rather than just exchange values. This paradigm shift is crucial for addressing current environmental challenges from a more comprehensive perspective. Furthermore, experts such as Sánchez et al. (2019) underscore the need to incorporate biodiversity and planetary boundaries into economic frameworks, highlighting environmental economics’ commitment to sustainability. This context of transformation and deeper awareness of the interconnection between economics and nature lay the groundwork for addressing current environmental challenges from an integrated perspective, where eco-innovation appears to play a crucial role [1, 2].

A concept that has increasingly become important in business practices is eco-innovation, also known as green innovation. While traditional corporate innovation often focuses on noticeable changes in products and processes aimed at generating economic value, eco-innovation emphasizes the need to address environmental and social concerns, thereby enhancing a company’s competitiveness. This concept recognizes the importance of meeting the needs of various stakeholders, who may sometimes have conflicting interests and goals [3]. Moreover, eco-innovation can be categorized into different types, including improvements in products, processes, and organizational systems, all of which aim to reduce or mitigate the adverse environmental impacts of business operations [4].

Regarding eco-innovation, it is proposed that larger companies are the most innovative as they have the necessary resources for accessing technologies and even for research and development [5]. However, SMEs constitute the majority of companies in developing countries, highlighting the importance of addressing eco-innovation for this group of companies, which is the subject of the present research [3].

Eco-innovation presents both significant challenges and benefits for small and medium enterprises (SMEs). A major challenge for SMEs lies in their limited financial and technical resources. Unlike larger corporations, SMEs often lack the capital to invest in advanced green technologies or to hire specialists with expertise in sustainability. This resource constraint can hinder their ability to adopt and implement eco-innovative solutions effectively. Additionally, regulatory complexities pose another barrier. SMEs operating in diverse regions must navigate an intricate web of local, national, and international environmental regulations, which can be time-consuming and costly. The lack of a unified regulatory framework complicates their compliance efforts and may discourage eco-innovation adoption. Furthermore, cultural resistance within organizations often delays or undermines eco-innovation initiatives. Employees and management accustomed to traditional practices may resist changes, particularly if the benefits are not immediately apparent [6, 7].

In the long term, eco-innovation can yield transformative impacts that underscore its sustainability benefits. Environmentally innovative SMEs contribute to a circular economy by designing products and services that minimize waste and encourage reuse, repair, and recycling. This shift reduces dependency on finite resources and ensures long-term economic viability. Furthermore, eco-innovation fosters resilience against environmental risks and regulatory changes. SMEs that proactively adopt sustainable practices are better equipped to adapt to stricter environmental regulations, fluctuating resource availability, and changing consumer preferences. From a social perspective, SMEs implementing eco-innovative solutions play a significant role in community development. They create green jobs, stimulate local economies, and raise awareness about sustainability, contributing to broader societal benefits [8–10].

Small and Medium Enterprises (SMEs) play a pivotal role in Colombia’s economy, contributing significantly to economic growth, job creation, innovation, and social development. Despite facing various challenges, SMEs remain integral to the Colombian economic landscape due to several factors. Firstly, SMEs are major contributors to job creation in Colombia. According to data from the National Administrative Department of Statistics (DANE), SMEs account for a significant portion of total formal employment in the country. This is particularly crucial in a country with high unemployment rates and a large informal sector. SMEs provide employment opportunities for a diverse range of individuals, including women, youth, and vulnerable populations, thereby fostering inclusive growth and reducing poverty levels [11].

Secondly, SMEs are important drivers of economic growth and innovation. Despite their size, SMEs in Colombia exhibit high levels of dynamism and entrepreneurial spirit. They often serve as incubators for new ideas, technologies, and business models, driving innovation across various sectors of the economy. Additionally, SMEs contribute to the diversification of Colombia’s industrial base, promoting competitiveness and resilience in the face of economic shocks [12].

Furthermore, SMEs play a crucial role in promoting regional development and reducing inequality. In Colombia, SMEs are dispersed across different regions, including rural and marginalized areas. By operating in these regions, SMEs stimulate local economies, create multiplier effects, and contribute to infrastructure development. Moreover, SMEs empower local communities by providing opportunities for entrepreneurship and skill development, thereby reducing regional disparities and promoting social cohesion [13].

Additionally, SMEs serve as key actors in Colombia’s export sector. While large multinational corporations dominate the country’s export market, SMEs play a complementary role by supplying goods and services to these larger firms, participating in global value chains, and accessing international markets. This not only enhances Colombia’s export competitiveness but also generates foreign exchange earnings, which are essential for economic stability and growth [14].

Eco-innovation, often referred to as green innovation, represents a transformative approach to innovation that prioritizes environmental sustainability alongside economic and social objectives. It involves the development and adoption of new processes, products, and organizational practices that reduce environmental impacts while enhancing resource efficiency and competitiveness [3, 4]. This concept is particularly relevant for small and medium enterprises (SMEs), as these organizations are pivotal drivers of regional economies but often face significant resource constraints that limit their capacity for innovation. This research focuses on the intersection of eco-innovation and social and economic performance within Colombian SMEs, as these dimensions directly address the immediate challenges and opportunities faced by these enterprises. While comprehensive sustainable performance encompasses environmental, social, and economic factors, the emphasis on social and economic performance reflects the pressing need for SMEs to enhance their financial viability and societal contributions in contexts characterized by limited resources and socio-economic disparities. By analyzing the economic and social outcomes of eco-innovation, this study aims to provide actionable insights that can support SMEs in achieving a balance between sustainability and economic resilience, especially in developing economies like Colombia [15, 16].

This research addresses a significant gap in understanding the role of eco-innovation within SMEs, particularly in the context of developing economies. While existing literature predominantly focuses on large firms, there is limited empirical evidence on how SMEs in these regions adopt and benefit from eco-innovation practices. Moreover, the study expands the scope by exploring the social and economic impacts of eco-innovation, moving beyond the traditional emphasis on environmental performance. This holistic approach is crucial, as SMEs in countries like Colombia contend with unique challenges such as limited resources, regulatory hurdles, and socio-economic inequalities.

Additionally, the use of structural equation modeling (SEM) to analyze these relationships contributes to methodological advancements by providing robust insights into the interconnections between eco-innovation, environmental performance, and social-economic outcomes. This approach enables the identification of nuanced dynamics that traditional studies may overlook. By focusing on SMEs—a significant segment of Colombia’s economic fabric—this research advances our understanding of how sustainability practices can drive inclusive growth and regional development, a topic underexplored in prior literature.

Consequently, our research question states: “What is the impact of eco-innovation and environmental performance on economic and social performance of small and medium enterprises in Colombia”. This study starts with an introduction, followed by an exhaustive literature review and the research model. The next chapter explains the methodology, followed by the results and discussion. The article finishes with the research limitations.

## Theoretical background

### Social and economic performance

Social and economic performance are two interrelated aspects of organizational success that address different dimensions of a company’s impact and effectiveness. Social performance refers to how well an organization fulfills its responsibilities to society, including its employees, customers, and the community. This involves ethical business practices, corporate social responsibility (CSR) initiatives, and contributions to social welfare, such as environmental sustainability and fair labor practices. According to Wood [17], social performance can be assessed through principles of social responsibility, processes of social responsiveness, and outcomes of social behavior.

Economic and social performance includes also a company’s ability to generate sustainable financial profits while simultaneously contributing to the well-being of its employees and society as a whole [18]. A key aspect of economic and social performance is the profitability of the company [19]. According to Barriga et al. [20], a company with a solid economic performance manages to generate consistent financial benefits over time. Larbi-Siaw et al. [21] assert that profitability is an important indicator of the economic success of a company, as it demonstrates its ability to generate income and be financially stable. Hojnik [22] also highlights the importance of profitability as a central aspect of economic and social performance.

Another significant factor in economic and social performance is the quality of the product or service and has a direct impact on customer satisfaction and, therefore, on the profitability of the company [20, 23]. According to these authors, offering high-quality products or services can generate greater customer loyalty and an increase in sales and financial benefits. On the other hand, job satisfaction is also an important aspect of economic and social performance and is positively related to employee performance and the overall success of the company [21]. Quality and job satisfaction are key elements for the economic and social performance of a company and companies should focus on creating shared value, that is, generating benefits for both the company and society [22, 24].

### Environmental performance

In terms of environmental performance, companies are required to implement eco-innovative practices and prioritize environmental sustainability. Environmental performance encompasses the strategies and measures a company employs to mitigate its negative environmental impact and enhance the conservation of natural resources [19].

Minimizing material consumption is essential for enhancing environmental performance. Studies have shown that companies adopting eco-innovation practices can notably decrease their use of materials and natural resources, resulting in both environmental and economic advantages [25]. Barriga et al. [20] argue that optimizing resource use and reducing waste generation are fundamental aspects of sustainable environmental performance. They also stress the need to control emissions and manage waste effectively to mitigate negative environmental impacts. Larbi-Siaw et al. [21] assert that stringent control over emissions and waste is critical for achieving sustainability. They further emphasize the importance of implementing recycling and product reuse strategies, which play a vital role in conserving resources and reducing the overall environmental impact.

Sustainable environmental performance encompasses practices such as waste reuse and recycling, which enable companies to reduce their environmental impact and adopt more sustainable operations. By minimizing waste, businesses not only decrease their environmental footprint but also lower production costs by reducing the need for new materials [22]. Additionally, sustainability practices in environmental performance involve adopting cleaner production technologies, implementing best available techniques, and enhancing energy efficiency. These measures help to reduce greenhouse gas emissions, conserve natural resources, and protect ecosystems. The literature highlights the crucial role of sustainable economic and social performance, alongside environmental performance, in achieving comprehensive sustainable development [23].

In recent years, the topic of sustainable performance has gained even more relevance in business management. Authors such as Cao [26] have proposed that implementing sustainable goals in workshop programming can provide a framework for decision-making in manufacturing companies. On the other hand, Yue et al. [27] have studied the relationship between Environmental Management Systems (EMS), Green Human Resource Management (Green HRM) practices, and Organizational Citizenship Behavior for the Environment (OCBE) as antecedents of the Triple Bottom Line (TBL) performance of manufacturers. Other authors studied corporate sustainability in individual SMEs from a Triple Bottom Line (TBL) perspective. They analyzed the relationship between sustainability practices and business outcomes in SMEs and how these companies can achieve a balance between the three dimensions of the TBL. In general, recent literature highlights the importance of eco-innovation and corporate sustainability for the long-term performance of companies, as well as the need to develop assessment frameworks and business models that integrate these considerations [28, 29].

Literature suggest that environmental performance increase competitive advantage and as a positive impact on different economic and social indicators [30–32]. High environmental performance reduces pollution, conserves resources, and minimizes waste, directly contributing to cost savings and operational efficiency, which enhances economic performance. This aligns with resource-based theory, which suggests that firms that efficiently utilize resources gain competitive advantages. Furthermore, environmental initiatives such as pollution control, renewable energy adoption, and eco-friendly products resonate with stakeholder expectations, building trust and goodwill. Social performance benefits as firms with strong environmental practices are seen as responsible corporate citizens, improving community relations and employee satisfaction [33, 34]. Consequently, the first hypothesis states.

**H1: Environmental performance has a positive impact on social and economic performance**

### Eco-innovation

Eco-innovation is widely recognized as a key pathway to achieving sustainable development from a business perspective. Its core elements include the focus of innovation, which pertains to different aspects of the company; the reduction of environmental impact through the efficient management of resource consumption, emissions, pollutants, and waste; a market-oriented approach that enhances competitiveness while considering stakeholder interests; and a commitment to sustainability that upholds the economic and social objectives of the company [4, 15]. However, definitions of eco-innovation vary significantly, often reflecting subtle differences based on the extent of environmental commitment adopted by an organization. A typical example is the following definition of eco-innovation given by the European Commission in 2007 within the "Competitiveness and Innovation Framework Programme" and referenced by Peyravi and Jakubavicius [35]:

"Any form of innovation as a significant and demonstrable progress towards the goal of sustainable development, through the reduction of environmental impacts or the achievement of a more efficient and responsible use of natural resources, including energy." (p. 4)

This definition highlights two critical aspects of the eco-innovation concept: its alignment with general innovation practices and its focus on reducing environmental impact. It also emphasizes the motivation behind eco-innovation, which is rooted in the political agenda of sustainable development. Moreover, international organizations such as the Organisation for Economic Co-operation and Development (OECD) assert that the defining characteristic of eco-innovation is its effectiveness in reducing environmental impact. According to the OECD, eco-innovation involves the creation or implementation of new or significantly improved products (including goods and services), processes, marketing strategies, organizational structures, and institutional arrangements that, whether intentional or not, lead to measurable improvements in environmental sustainability compared to existing alternatives [36, 37].

The OECD’s definition implies that the motivation for eco-innovation may extend beyond environmental concerns, such as reducing costs associated with waste management. Additionally, the definition emphasizes the importance of demonstrating improvement over existing alternatives. This comparative aspect is crucial, as it requires evaluation against both intra- and inter-organizational options, making the concept relative and time-bound—a significant consideration for SMEs given their unique characteristics. Moreover, the OECD highlights that eco-innovation can be both technological and non-technological, encompassing changes in organizational structure or marketing strategies, which is particularly relevant for SMEs [37, 38].

Eco-innovation is considered as a significant fac tor in avoiding environmental damage. Reducing waste and better usage of resources through innovation enhance environmental performance if the organization [39–41]. Eco-innovation involves adopting new processes, technologies, or practices that aim to reduce environmental harm while maintaining economic viability. By its very nature, eco-innovation prioritizes sustainability goals, directly targeting improvements in environmental performance. For instance, innovations in energy efficiency, waste reduction, and water conservation help reduce greenhouse gas emissions and resource depletion. According to the Resource-based view theory (RBV), eco-innovations can be unique resources that provide firms with capabilities to manage environmental risks effectively. Moreover, institutional theory highlights the role of external pressures—such as regulatory frameworks, market demands, and societal expectations—in driving eco-innovation. Firms adopting eco-innovation not only meet compliance requirements but also often exceed them, resulting in measurable improvements in environmental performance, such as reduced carbon footprints or improved biodiversity conservation [8]. Therefore, the second hypothesis states that:

**H2: Eco-innovation has a positive impact on environmental performance**

On the same, eco-innovation also has leads to improved and more efficient processes and also reduces pollution on environment, enhancing societies wellbeing [39, 42, 43]. The rationale for this hypothesis stems from stakeholder theory, which posits that businesses must address the needs and concerns of various stakeholders—including employees, customers, investors, and society at large—for long-term success. Eco-innovation enhances social performance by addressing societal concerns related to environmental degradation and fostering inclusive growth. From an economic perspective, eco-innovation aligns with the dynamic capabilitie´s framework, which emphasizes a firm’s ability to adapt to changing environments. Firms that embrace eco-innovation can create value by accessing new markets, reducing costs through efficient resource use, and enhancing brand reputation. Additionally, the competitive advantage gained through eco-innovation often results in increased revenues and market share. Eco-innovation’s dual focus on sustainability and profitability ensures that social well-being and economic growth are complementary outcomes, reinforcing the hypothesis [44]. Therefore, the third hypothesis proposes that:

**H3: Eco-innovation has a positive impact on social and economic performance**

## Methodology

This empirical investigation focuses on small and medium enterprises (SMEs) in Colombia, a country characterized by its significant economic reliance on SMEs, which account for a substantial proportion of employment and GDP. Colombia’s socio-economic and environmental challenges, including limited financial resources, regulatory constraints, and regional inequalities, make it an ideal context to explore the adoption and impact of eco-innovation. These enterprises operate in a dynamic environment where balancing profitability with sustainability is essential, particularly in light of global shifts toward greener economies and stricter environmental standards. Understanding the intersection of eco-innovation and SME performance in Colombia contributes valuable insights into fostering sustainability in resource-constrained settings. The research methodology employed herein is confirmatory factor analysis, chosen for its capacity to afford the investigator heightened flexibility in formulating hypotheses concerning the structure of the construct. The objective is to scrutinize and validate extant theories and relationships [45]. Given the accelerated maturation of web-based survey methods, widely adopted by academic and commercial researchers for online data collection [46], an electronic survey instrument was developed and disseminated through a dedicated website between 2022 and 2023). The survey link was subsequently distributed to targeted respondents via email. Data collection commenced on 01/07/2022 and concluded on 31/12/2023. At the beginning of the survey, participants were informed that they could withdraw from the survey at any stage. Informed consent was obtained from all participants. The consent was considered given when participants chose to continue with the survey after being informed of their right to withdraw at any stage. The survey was approved by the Ethics committee of Colegio de Estudios Superiores de Administración–CESA.

The universe comprised 5,627 SMEs within the region, thereby ensuring a comprehensive representation of the target population. From this, a sample of 568 SMEs, representing 10.09% of the total SME population, participated in the survey. Only fully and correctly completed surveys were included in the sample, ensuring high data quality and validity. This methodological approach demonstrates strict adherence to rigor, as incomplete responses were excluded, thereby eliminating potential biases associated with missing data.

The questionnaire, employing a five-point Likert scale anchored at two extremes (Completely Agree—Completely Disagree), was designed to assess variables at an ordinal measurement level. It featured a structured set of statements about operationalizing the model’s variables, divided into two sections: Section A, with demographic information, and Section B, focusing on the variables. The survey was directed at various personnel, including managers, directors, administrative staff, operational staff, and service personnel, to capture diverse perspectives, perceptions, and expectations associated with organizational innovation.

The eco-innovation section of the questionnaire was based on the works of Barriga et al. [20] and Hojnik [22]. Social and economic performance section instrument, as well as the environmental performance section considered the works of Baumgartner & Rauter [4] and Hojnik [22].

**Fig 1:**
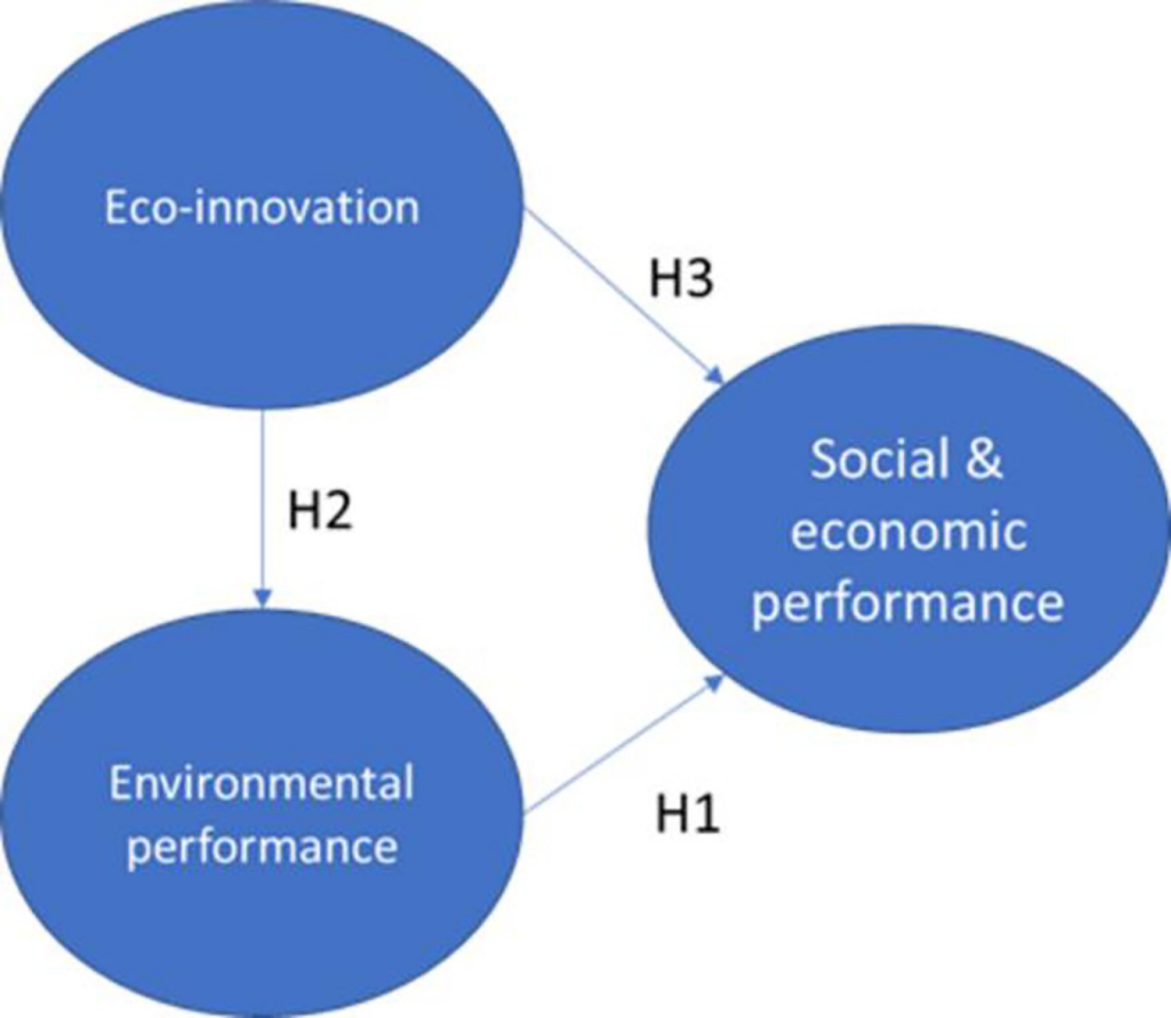
Hypothesized model comes here. Source: By the authors.

The study, employs structural equation modeling to evaluate relationships between small and medium-sized enterprises (SMEs). Statistical analysis encompassed the utilization of both SPSS V21 and Structural Equation Modeling (SEM) via Analysis of Moment Structures (AMOS). Those analytical instruments facilitated a comprehensive examination of the dataset, validating the conceptual model delineated in Fig 1, estimating predictive relationships among variables, assessing model fit indices, and determining confidence levels.

Confirmatory Factor Analysis (CFA) was employed to analyze the relationships between observed continuous latent variables and evaluate the overall fit of the measurement model, drawing upon the methodology outlined by Hair et al. [47]. The estimation of factor loadings, serving as indicators of the strength of the relationship between each observed variable and its underlying latent construct (excluding cross-loadings), was also undertaken. The intercorrelation of latent constructs, akin to oblique rotation in exploratory factor analysis, was conducted. Internal consistency was evaluated using Cronbach’s alpha coefficient and examining total item-to-construct correlations (Table 1). All values are above 0,7, showing a high degree of consistency.

**Table 1:** Cronbachs´s alpha.

Variable	Cronbach´s Alpha
Eco-innovation	0,812
Social and economic performance	0,824
Environmental performance	0,827

Source: By the authors

Table 2 presents the coefficient values for the constructs. All constructs exhibited values above 0.7, surpassing the established cutoff level for basic research [48, 49].

**Table 2:** Baseline comparison.

Model	NFI Delta1	RFI rho1	IFI Delta2	TLI rho2	CFI	GFI
Default model	,988	,982	1,000	1,000	1,000	,991
Saturated model	1,000		1,000		1,000	1,000
Independence model	,000	,000	,000	,000	,000	,509

Source: By the authors

Goodness-of-fit indices (GFI) were employed to support the model. The model comprises 45 distinct sample moments with 21 unique parameters to estimate. The Chi-square value is equal to 24.171 with 24 degrees of freedom, resulting in a CMIN/DF of 1.007 and a probability level of 0.000. It should be noted that Wheaton et al. [50] suggested a ratio of approximately five or less as a reasonable criterion, and Carmines & McIver [51] suggested ratios in the range of 2:1 or 3:1 as indicators of an acceptable fit between the hypothetical model and the sample data.

The Comparative Fit Index (CFI) value above 0.9 is consistent with the model, yielding a result of 1.00 [48]. Furthermore, the reliability of each construct in the model was assessed using various fit statistics, and the Root Mean Square Error of Approximation (RMSEA) was deemed acceptable, with the model having a value of 0.004 (Table 3), well below the maximum threshold of 0.08 [52–54]. Comparisons of fit reference indices suggest that the hypothetical model fits well with the observed variance-covariance matrix in relation to the null or independence model. Additionally, average variance extracted (AVE) and composite reliability were used to assess the model validity. Values above 0,5 for AVE indicator and above 0,7 (CR), showed in Table 4, indicate a robust model [47].

**Table 3:** RMSEA.

Model	RMSEA	LO 90	HI 90	PCLOSE
Default model	,004	,000	,034	,999
Independence model	,312	,300	,323	,000

Source: By the authors

**Table 4:** Composite reliability (CR) & average variance extracted (AVE).

Factor	CR	AVE
Ecoinnovation	0,815	0,597
Environmental performance	0,827	0,616
Social and economic performance	0,832	0,625

Source: By the authors

## Results

**Fig 2:**
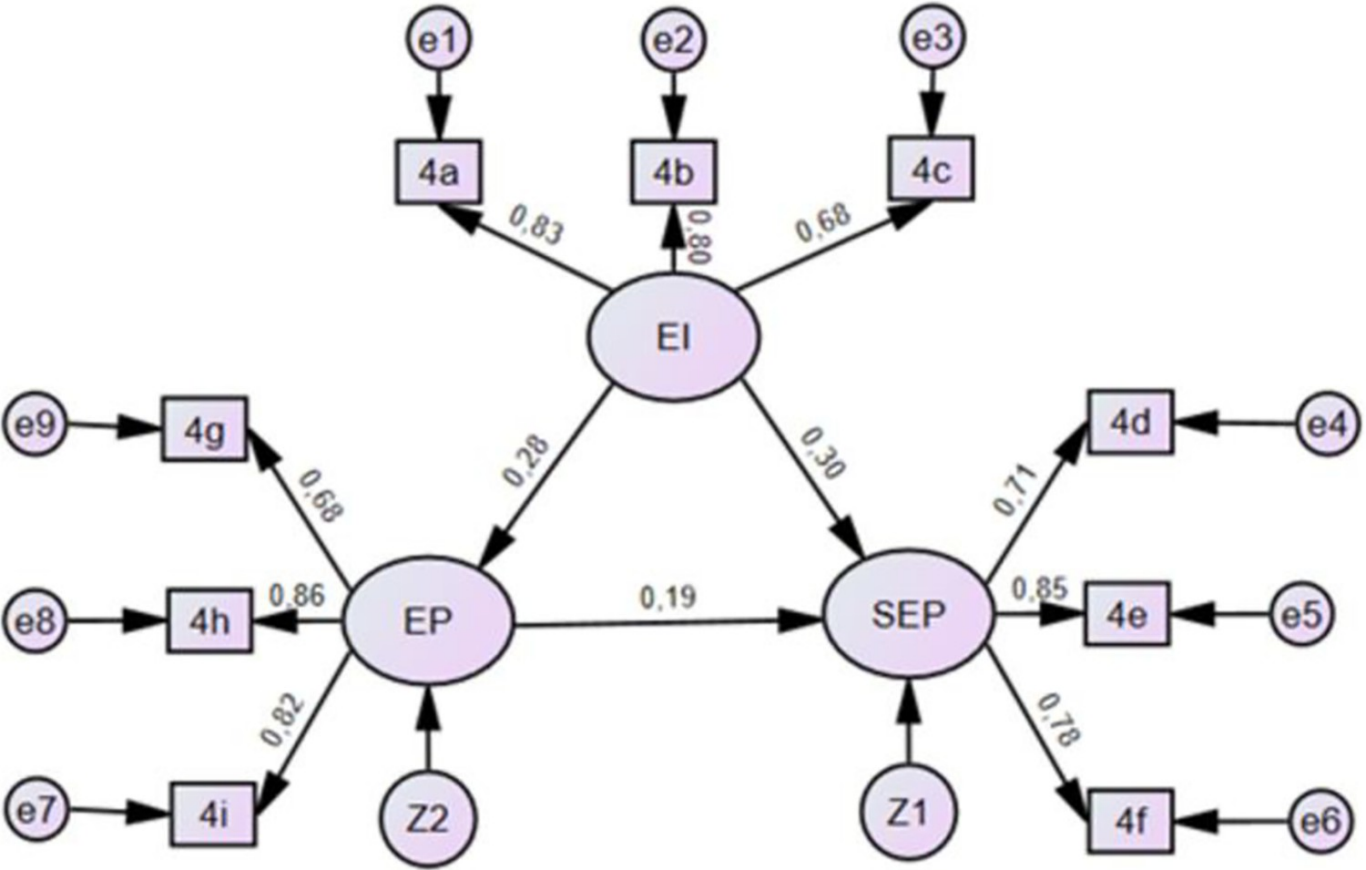
SEM Model comes here. Source: By the authors.

Fig 2 show the hypothesized model with results.

The structural equation modeling (SEM) analysis results presented in Table 5 indicate significant relationships among the latent constructs of social and economic performance (DSE), environmental performance (DA), and eco-innovation (EI).

**Table 5:** Results with standardized values.

			Estimate	S.E.	C.R.	P label	
Environmental performance (EP)	→	Social and economic performance (SEP)	,172	,046	3,723	***	H1
Eco-innovation (EI	→	Environmental performance (EP)	,298	,053	5,656	***	H2
Eco-innovation (EI	→	Social and economic performance (SEP)	,285	,051	5,621	***	H3

Source: By the authors

Firstly, the path from environmental performance (DA) to social and economic performance (DSE) shows a positive and significant relationship (estimate = 0.172, S.E. = 0.046, C.R. = 3.723, p < 0.001), suggesting that improvements in environmental performance are associated with enhanced social and economic performance. This finding aligns with the growing recognition of the interconnectedness between environmental sustainability and economic prosperity, indicating that organizations or entities with stronger environmental performance tend to exhibit better social and economic outcomes.

Secondly, the path from environmental performance (DA) to eco-innovation (EI) demonstrates a strong positive relationship (estimate = 0.298, S.E. = 0.053, C.R. = 5.656, p < 0.001), indicating that higher levels of environmental performance are associated with greater eco-innovation. This result suggests that organizations or entities with a stronger commitment to environmental sustainability are more likely to engage in innovative practices aimed at reducing environmental impact or promoting sustainability across their operations.

Thirdly, the path from eco-innovation (EI) to social and economic performance (DSE) also shows a significant positive relationship (estimate = 0.285, S.E. = 0.051, C.R. = 5.621, p < 0.001), indicating that eco-innovation contributes positively to social and economic performance. This finding highlights the importance of innovation, particularly in the context of environmental sustainability, as a driver of overall organizational performance. Organizations that prioritize eco-innovation are likely to experience benefits not only in terms of environmental outcomes but also in social and economic dimensions.

The results of the analysis results underscore the intricate relationships among environmental performance, eco-innovation, and social and economic performance. The findings suggest that efforts to improve environmental performance and foster eco-innovation can lead to positive outcomes across multiple dimensions of organizational performance. These insights have important implications for policymakers, managers, and other stakeholders interested in promoting sustainability and innovation within organizations, as they highlight the potential synergies between environmental initiatives and broader organizational goals. However, further research may be needed to explore additional factors that could influence these relationships and to assess the generalizability of these findings across different contexts or industries.

## Discussion

Eco-innovation fosters positive social outcomes by addressing societal concerns and contributing to sustainable development. By prioritizing eco-friendly practices, organizations demonstrate their commitment to corporate social responsibility (CSR), which enhances their reputation and stakeholder trust [55]. Furthermore, eco-innovation often leads to the creation of green jobs and opportunities for skill development, thus promoting social inclusivity and economic empowerment within communities [56]. By engaging with stakeholders and aligning their objectives with broader societal goals, eco-innovative organizations can effectively address social challenges while driving positive change.

Also, Eco-innovation plays a pivotal role in advancing CSR objectives by aligning organizational strategies with societal needs and expectations. Research indicates that eco-innovative practices contribute to the fulfillment of CSR commitments, enhancing organizational reputation and stakeholder trust [55]. By integrating environmental considerations into their operations and products, organizations demonstrate their commitment to sustainable development and responsible business practices. This alignment between eco-innovation and CSR not only enhances social performance but also fosters long-term sustainability and competitiveness.

Additionally, Eco-innovation contributes to community development by creating opportunities for economic empowerment, job creation, and sustainable livelihoods. Research suggests that eco-innovative practices often lead to the emergence of green industries and the development of local supply chains [57]. By investing in renewable energy, waste management, and sustainable agriculture, organizations can stimulate economic growth while safeguarding environmental resources. Furthermore, eco-innovative initiatives, such as green infrastructure projects and eco-friendly urban planning, can enhance the quality of life in communities and promote social inclusion [58]. By prioritizing the needs of local stakeholders and fostering collaboration, eco-innovative organizations can contribute to the social and economic development of the regions in which they operate.

On the other hand, the environmental benefits of eco-innovation are profound and multifaceted. Through the adoption of sustainable technologies and processes, organizations can reduce their ecological footprint, minimize resource consumption, and mitigate pollution [59]. Eco-innovation encourages the development of cleaner production methods, renewable energy solutions, and waste reduction strategies, thereby contributing to biodiversity conservation and climate change mitigation [57]. By embracing eco-innovation, organizations play a pivotal role in transitioning towards a more sustainable and resilient future, safeguarding natural ecosystems and ensuring the well-being of current and future generations.

One of the primary benefits of eco-innovation is its ability to improve resource efficiency within organizations. By optimizing processes, products, and services, eco-innovative practices enable organizations to minimize resource consumption and waste generation [59]. Research suggests that eco-innovation leads to significant improvements in resource productivity, allowing organizations to achieve more with fewer inputs [60]. Through the adoption of technologies such as energy-efficient machinery, closed-loop systems, and sustainable materials, organizations can reduce their reliance on finite resources and enhance environmental performance. By prioritizing resource efficiency through eco-innovation, organizations can achieve cost savings, enhance competitiveness, and reduce their environmental footprint.

Another important discussion is the contribution of eco-innovation in addressing climate change, which is one of the most pressing environmental challenges of our time. Eco-innovation plays a critical role in advancing climate change mitigation efforts. By developing low-carbon technologies, renewable energy solutions, and carbon capture technologies, organizations can reduce their greenhouse gas emissions and contribute to global efforts to combat climate change [55]. Research suggests that eco-innovation leads to significant reductions in carbon emissions and enhances organizational resilience to climate-related risks [61]. By investing in climate-friendly technologies and practices, organizations can align their operations with climate goals and regulatory requirements, positioning themselves for long-term sustainability and competitiveness.

Contrary to the misconception that sustainability incurs additional costs, eco-innovation has been shown to yield substantial economic benefits for organizations. By improving resource efficiency and operational effectiveness, eco-innovation enhances cost savings and competitiveness [60]. Moreover, eco-innovative products and services often cater to a growing market demand for sustainable solutions, opening up new revenue streams and market opportunities [58]. Additionally, eco-innovation fosters innovation and enhances organizational resilience by encouraging adaptability to changing market conditions and regulatory requirements [62]. Ultimately, organizations that embrace eco-innovation stand to achieve long-term profitability and sustainable growth. Examples from Colombian SMS´s illustrate the possible benefits implementing eco-innovation initiatives. Altough Colombian organizations focus mainly in pollution prevention, product stewardship, clean technology, and sustainable vision focused on the base-of-the-pyramid, other successful case are SME´s in the construction sector that have integrated circular economy principles by emphasizing resource recovery, the use of green teams, and strong management commitment. These eco-innovative strategies not only contribute to environmental sustainability but also improve the company’s cost-efficiency, showcasing how sustainable practices can drive both environmental and economic performance. Similarly, a Colombian coffee producer, utilizes organic fertilizers and renewable energy, aligning with eco-certifications such as Rainforest Alliance. These practices help conserve resources while enhancing the brand’s appeal in the global market, demonstrating the commercial advantages of eco-innovation [63].

Eco-innovation offers organizations opportunities to reduce costs and enhance efficiency through resource optimization, waste reduction, and process improvements. Research indicates that eco-innovative practices lead to significant savings in energy, water, and raw materials, thereby reducing production costs and enhancing profitability [60]. By adopting cleaner production methods and embracing sustainable technologies, organizations can minimize waste generation, streamline operations, and achieve higher levels of productivity. These cost-saving benefits not only contribute to short-term financial gains but also enhance organizational resilience and competitiveness in the long run.

Additionally, Eco-innovation provides organizations with a strategic advantage by positioning them as leaders in sustainability and responsible business practices. Research suggests that eco-innovative firms enjoy enhanced brand reputation, customer loyalty, and stakeholder trust, which translate into sustained competitive advantage [16]. By proactively addressing environmental concerns and demonstrating commitment to sustainability, organizations can differentiate themselves from competitors, attract investors, and strengthen their market position. Furthermore, eco-innovation fosters a culture of continuous improvement and adaptive capacity, enabling organizations to respond effectively to evolving market dynamics and regulatory requirements.

The findings of the provided research contribute significantly to the eco-innovation literature by confirming the positive impact of eco-innovation on environmental, social, and economic performance within SMEs in Colombia. These results align with existing studies emphasizing that eco-innovation enhances resource efficiency, reduces waste, and fosters environmental sustainability [8, 60]. Similar to previous research, the study highlights that eco-innovation improves social outcomes through job creation and community development, consistent with findings by Delmas and Pekovic [56] on the societal benefits of sustainable practices.

Additionally, the research validates the role of eco-innovation in driving economic performance, particularly by enhancing profitability and market competitiveness, as observed in studies by Hojnik [22] and Horbach et al. [58]. However, this study uniquely contributes by focusing on Colombian SMEs, a group often underrepresented in global research, thus addressing a critical contextual gap. It reinforces the notion that eco-innovation is essential for fostering inclusive economic growth in developing economies, echoing the conclusions of Valdez-Juárez & Castillo-Vergara [7] regarding SMEs in Latin America.

The findings also align with existing literature on eco-innovation in developing countries, highlighting both the opportunities and challenges faced by small and medium enterprises (SMEs) in these regions. The study confirms that eco-innovation positively influences environmental performance by enhancing resource efficiency and reducing waste, which is consistent with studies conducted in developing countries such as Turkey and China. For instance, Yurdakul and Kazan [39] found that eco-innovative practices in Turkish manufacturing SMEs significantly improved environmental and economic outcomes, emphasizing the critical role of resource optimization. Similarly, Cai and Li [8] highlighted the environmental and competitive advantages of eco-innovation in Chinese SMEs, particularly in addressing sustainability challenges unique to developing economies.

Moreover, the research supports the conclusion that eco-innovation drives economic and social performance, including profitability and community development. These findings resonate with studies in Latin America, such as Valdez-Juárez & Castillo-Vergara [7], who demonstrated that eco-innovation fosters resilience and market competitiveness among SMEs in resource-constrained environments. Additionally, the study’s focus on job creation and local development aligns with Delmas and Pekovic’s [56] work, which emphasized the social inclusivity benefits of sustainable innovation in emerging markets.

However, the findings also underscore challenges unique to developing countries, such as limited financial and technical resources, regulatory complexities, and socio-economic disparities. These barriers are widely recognized in the literature as significant constraints to the adoption of eco-innovation, as noted by Munodawafa and Johl [44]. The study further expands on this by providing a Colombian perspective, adding depth to the broader discourse on how regional factors influence eco-innovation’s adoption and impact.

## Theoretical implications

The theoretical implications of the study on eco-innovation and its impact on SMEs in Colombia contribute significantly to existing literature by advancing our understanding of the interconnectedness between sustainability practices and organizational performance. The findings reinforce established frameworks, such as the resource-based view (RBV) and stakeholder theory, while offering nuanced insights specific to the context of SMEs in developing economies. The study validates RBV’s assertion that eco-innovation serves as a strategic resource, enabling firms to leverage unique capabilities for managing environmental risks and gaining competitive advantage. This aligns with the premise that sustainable practices, including resource optimization and waste reduction, are not merely compliance mechanisms but also value-creating strategies that bolster long-term organizational resilience.

From a stakeholder perspective, the study substantiates the idea that businesses addressing environmental and social concerns foster stronger relationships with stakeholders, including customers, employees, and regulators. By integrating eco-innovation into their operations, SMEs can meet diverse stakeholder expectations, enhancing their legitimacy and trustworthiness. The research highlights how eco-innovation’s emphasis on aligning organizational goals with societal needs contributes to broader social and economic outcomes, offering empirical support for stakeholder theory in the sustainability domain.

The use of structural equation modeling (SEM) in the study introduces methodological rigor to the exploration of eco-innovation’s multidimensional impact. The approach provides robust evidence of the interdependencies between environmental, social, and economic performance, advancing theoretical discourse on the triple bottom line (TBL). The findings emphasize the importance of adopting integrated frameworks for analyzing sustainability, where environmental initiatives are not viewed in isolation but as catalysts for economic growth and social development.

Additionally, the study addresses a critical gap in the literature by focusing on SMEs in a developing economy, challenging the traditional emphasis on large firms in global sustainability research. This context-specific analysis reveals unique challenges—such as resource constraints and regulatory complexities—that shape the adoption and efficacy of eco-innovation in smaller enterprises. It underscores the necessity of adapting existing theoretical models to account for the socio-economic and institutional realities of developing countries.

## Practical implications

Eco-innovation represents a significant opportunity for local organizations in Colombia to address pressing societal and environmental challenges while enhancing their competitive advantage. By integrating eco-friendly practices into their operations, these organizations can demonstrate a strong commitment to corporate social responsibility (CSR), thereby enhancing their reputation and building trust with stakeholders. Research by Schaltegger and Wagner [55] underscores that such initiatives not only fulfill CSR commitments but also foster long-term sustainability and competitiveness. This approach can position organizations as leaders in responsible business practices, attracting investors and strengthening their market position.

One of the primary benefits of eco-innovation is its potential to create green jobs and opportunities for skill development, promoting social inclusivity and economic empowerment within communities. By investing in renewable energy projects, sustainable agriculture, and waste management initiatives, organizations can stimulate local economies and provide sustainable livelihoods. Delmas and Pekovic [56] highlight that eco-innovation contributes significantly to community development by fostering the growth of green industries and local supply chains. This, in turn, can enhance the quality of life in communities and promote social inclusion, making a tangible impact on local development.

Engaging with stakeholders and aligning organizational objectives with broader societal goals is crucial for the success of eco-innovative initiatives. By hosting community forums and stakeholder meetings, organizations can ensure that their projects address the needs and concerns of local populations. This collaborative approach not only builds trust but also drives positive change by effectively addressing social challenges. The research indicates that eco-innovative organizations are better positioned to achieve social performance goals and foster long-term sustainability.

Reducing the environmental footprint is another critical aspect of eco-innovation. Organizations can achieve this by adopting sustainable technologies and processes that minimize resource consumption and mitigate pollution. Investments in energy-efficient machinery, closed-loop systems, and sustainable materials can lead to significant reductions in ecological impact. Such practices contribute to biodiversity conservation and climate change mitigation, safeguarding natural ecosystems for future generations. By prioritizing resource efficiency, organizations can achieve cost savings, enhance operational efficiency, and reduce their environmental footprint, improve environmental performance and leading to long-term benefits [59].

Eco-innovation also plays a pivotal role in advancing climate change mitigation efforts. By developing and implementing low-carbon technologies and renewable energy solutions, organizations can significantly reduce their greenhouse gas emissions. This aligns with global efforts to combat climate change and positions organizations to meet regulatory requirements. Eco-innovation not only reduces carbon emissions but also enhances organizational resilience to climate-related risks. This proactive approach can ensure that organizations remain competitive and sustainable in the face of evolving environmental challenges [55].

Contrary to the misconception that sustainability incurs additional costs, eco-innovation has been shown to yield substantial economic benefits. By improving resource efficiency and operational effectiveness, organizations can realize significant cost savings. Eco-innovative practices lead to significant improvements in resource productivity, enabling organizations to achieve more with fewer inputs. Additionally, eco-innovative products and services often cater to a growing market demand for sustainable solutions, opening up new revenue streams and market opportunities. This not only enhances profitability but also fosters long-term growth and resilience [60].

Moreover, eco-innovation provides organizations with a strategic advantage by positioning them as leaders in sustainability. By proactively addressing environmental concerns and demonstrating a commitment to sustainable development, organizations can enhance their brand reputation, customer loyalty, and stakeholder trust. Eco-innovative firms enjoy a sustained competitive advantage due to their strong market positioning. This reputation can attract investors and customers who prioritize sustainability, further strengthening the organization’s market position [16].

Fostering a culture of continuous improvement and adaptability is essential for organizations to thrive in the dynamic market environment. Eco-innovation encourages organizations to remain flexible and responsive to changing market conditions and regulatory requirements. Müller and Kolk [62] suggest that organizations embracing eco-innovation are better equipped to adapt to new challenges and seize emerging opportunities. This adaptability enhances organizational resilience, ensuring long-term sustainability and competitiveness.

From a broader perspective, eco-innovation addresses pressing global challenges, such as climate change and resource scarcity. By driving the development of low-carbon technologies, renewable energy solutions, and pollution mitigation strategies, eco-innovation plays a critical role in advancing climate change mitigation and biodiversity conservation. Businesses that proactively adopt eco-innovation not only contribute to global sustainability goals but also position themselves as leaders in environmental responsibility, enhancing their long-term resilience and market relevance.

Moreover, eco-innovation promotes collaboration between businesses, governments, and communities, creating a synergy that amplifies its impact. Collaborative eco-innovation initiatives, such as public-private partnerships and community engagement programs, ensure that the benefits of sustainability practices are equitably distributed across stakeholders. This fosters a shared sense of responsibility and encourages broader adoption of sustainable practices.

In essence, the broader implications of eco-innovation extend well beyond individual businesses. By addressing systemic issues such as inequality, environmental degradation, and economic instability, eco-innovation emerges as a cornerstone for sustainable development worldwide. It offers a replicable model for countries and regions seeking to integrate sustainability into their economic frameworks, ensuring that growth is inclusive, resilient, and environmentally sound. As demonstrated by the Colombian SMEs in the study, eco-innovation is not merely a pathway to better business performance but a transformative approach to achieving long-term societal and ecological well-being.

## Conclusion

This research provides a comprehensive examination of the role of eco-innovation in enhancing the environmental, social, and economic performance of small and medium enterprises (SMEs) in Colombia. By employing structural equation modeling (SEM) to analyze data from 568 SMEs, the study sheds light on how eco-innovation contributes to sustainability in resource-constrained environments. The findings validate the interconnectedness of environmental initiatives and organizational performance, reinforcing the importance of eco-innovation as a critical driver of sustainable development.

The research reveals that eco-innovation positively impacts environmental performance by optimizing resource efficiency, minimizing waste, and reducing emissions. This, in turn, enhances social and economic performance by improving profitability, product quality, job satisfaction, and community well-being. These insights align with the triple bottom line (TBL) framework, highlighting the interplay between environmental, economic, and social dimensions of sustainability. By adopting eco-innovative practices, SMEs not only fulfill regulatory requirements but also position themselves as leaders in sustainability, gaining competitive advantages in an increasingly eco-conscious global market.

The study’s theoretical implications emphasize the need to expand traditional sustainability frameworks to account for the unique challenges and opportunities faced by SMEs in developing economies. It advances the resource-based view (RBV) by demonstrating how eco-innovation acts as a strategic resource for achieving competitive advantage. Furthermore, it integrates stakeholder theory by showing that organizations that address societal and environmental concerns build stronger relationships with key stakeholders, fostering trust, legitimacy, and long-term resilience. The use of SEM enhances methodological rigor, offering robust evidence of the multifaceted impacts of eco-innovation on organizational performance.

In practical terms, the research underscores the transformative potential of eco-innovation for SMEs in Colombia and beyond. It demonstrates that sustainable practices are not merely ethical obligations but strategic imperatives that drive profitability, enhance brand reputation, and contribute to community development. By creating green jobs, fostering local economies, and aligning with global sustainability goals, SMEs can achieve inclusive growth while addressing pressing environmental challenges such as climate change and resource scarcity. These findings provide actionable insights for policymakers, encouraging them to develop targeted support mechanisms—such as financial incentives, capacity-building programs, and streamlined regulations—to foster eco-innovation in SMEs.

However, the study also highlights significant barriers to eco-innovation, including limited financial and technical resources, regulatory complexities, and cultural resistance to change. These challenges are particularly pronounced in developing economies like Colombia, where SMEs operate within a context of socio-economic disparities and institutional constraints. Addressing these barriers requires a concerted effort from businesses, governments, and international organizations to create enabling environments for sustainability.

The research makes a valuable contribution by focusing on Colombian SMEs, a group often underrepresented in global sustainability studies. While the findings are context-specific, they offer broader implications for emerging economies, emphasizing the replicability of eco-innovation strategies in resource-constrained settings. Future research could build on this work by exploring longitudinal impacts, incorporating qualitative insights, and examining cross-sectoral variations in eco-innovation adoption.

## Limitations

This study is not without its limitations. First, the use of a convenience sample introduces the potential for selection bias, as respondents were intentionally chosen based on their involvement in innovation management. Second, the study relies solely on survey responses for data collection. While the survey was meticulously designed to capture diverse perspectives, the self-reported nature of the data introduces the possibility of response bias. Respondents may have overstated their engagement in eco-innovative practices or their associated benefits to align with socially desirable norms or expectations. Additionally, Colombia is a country with a very low level of innovation as presented in World Economic Forum reports. Although this provides valuable insights specific to the Colombian context, the applicability of the results to other regions or countries with differing economic, regulatory, and cultural environments may be limited. The unique socio-economic and institutional dynamics in Colombia could influence the adoption and impact of eco-innovation differently compared to other settings. Therefore, generalization is questionable.

Future research should address these limitations by employing randomized sampling techniques, incorporating additional qualitative data, expanding the geographic scope, and conducting longitudinal studies to validate and extend the current findings.

## References

[1] Valencia J, Rodríguez JM, Mendoza JJA, Castaño JM. Valoración de los servicios ecosistémicos de investigación y educación como insumo para la toma de decisiones desde la perspectiva de la gestión del riesgo y el cambio climático. Luna Azul. 2017;(45):11–41.

[2] Sánchez J, Domínguez R, León M, Samaniego J, Sunkel O. Recursos naturales, medio ambiente y sostenibilidad: 70 años de pensamiento de la CEPAL: Cepal; 2019.

[3] Borsatto JMLS Bazani CL. Green innovation and environmental regulations: A systematic review of international academic works. Environmental science and pollution research. 2021:1–18. doi: 10.1007/s11356-020-11379-7 33141379

[4] Baumgartner RJ, Rauter R. Strategic perspectives of corporate sustainability management to develop a sustainable organization. Journal of Cleaner Production. 2017;140:81–92.

[5] Vieira AP, Radonjič G. Disclosure of eco-innovation activities in European large companies’ sustainability reporting. Corporate Social Responsibility and Environmental Management. 2020;27(5):2240–53. doi: 10.1002/csr.1961

[6] Klewitz J, Hansen EG. Sustainability-oriented innovation of SMEs: a systematic review. Journal of cleaner production. 2014;65:57–75.

[7] Valdez-Juárez LE, Castillo-Vergara M. Technological capabilities, open innovation, and eco-innovation: Dynamic capabilities to increase corporate performance of SMEs. Journal of Open Innovation: Technology, Market, and Complexity. 2021;7(1):8.

[8] Cai W, Li G. The drivers of eco-innovation and its impact on performance: Evidence from China. Journal of cleaner production. 2018;176:110–8.

[9] Geng D, Lai K-h, Zhu Q. Eco-innovation and its role for performance improvement among Chinese small and medium-sized manufacturing enterprises. International Journal of Production Economics. 2021;231:107869.

[10] Majid S, Zhang X, Khaskheli MB, Hong F, King PJH, Shamsi IH. Eco-efficiency, environmental and sustainable innovation in recycling energy and their effect on business performance: evidence from European SMEs. Sustainability. 2023;15(12):9465.

[11] Jauhari H, Periansya P. Economic growth, poverty, urbanization, and the Small and Medium Enterprises (SMEs) in Indonesia: Analysis of Cointegration and Causality. Binus Business Review. 2021;12(2):143–50.

[12] Díaz JVB. Sustainability and Competitiveness in SMEs in the Apparel Sector. Handbook of Research on International Business and Models for Global Purpose-Driven Companies: IGI Global; 2021. p. 404–30.

[13] Vanoni G, Omaña A. Female entrepreneurship and evolution in smes in the fashion system in Colombia. Compendium: Cuadernos de Economía y Administración. 2021;8(2):132–44.

[14] Bayon M, Aguilera P. Managerial perceptions of the strategic relevance of resources and capabilities and its configuration for firm competitiveness: an exploratory study. Competitiveness Review: An International Business Journal. 2021;31(3):462–76.

[15] Bucheli JM, Santa R, Tegethoff T, Quintero K. The Mediating Role of Eco-Innovation between Adaptive Environmental Strategy, Absorptive Capacity, and Environmental Performance. Sustainability. 2024;16(15):6504.

[16] Mady K, Battour M, Aboelmaged M, Abdelkareem RS. Linking internal environmental capabilities to sustainable competitive advantage in manufacturing SMEs: The mediating role of eco-innovation. Journal of Cleaner Production. 2023;417:137928.

[17] Wood DJ. Corporate social performance revisited. Academy of management review. 1991;16(4):691–718.

[18] Pazienza M, de Jong M, Schoenmaker D. Clarifying the concept of corporate sustainability and providing convergence for its definition. Sustainability. 2022;14(13):7838.

[19] Henríquez-Calvo L, Díaz-Martínez K, Chang-Muñoz EA, Guarín-García AF, Portnoy I, Ramírez JA. Analysis of the Impact Process Innovation and Collaboration on Competitiveness in Small and Medium-sized Enterprises: A Case Study in Colombia. Procedia Computer Science. 2024;231:636–41.

[20] Barriga Medina HR, Guevara R, Campoverde RE, Paredes-Aguirre MI. Eco-innovation and firm performance: Evidence from South America. Sustainability. 2022;14(15):9579.

[21] Larbi-Siaw O, Xuhua H, Donkor DO. Attaining sustainable business performance via eco-innovation under ecological regulatory stringency and market turbulence. Journal of Cleaner Production. 2023;394:136404.

[22] Hojnik J. In pursuit of eco-innovation: drivers and consequences of eco-innovation at firm level: Založba Univerze na Primorskem, = University of Primorska Press; 2017.

[23] Calik E, Bardudeen F. A measurement scale to evaluate sustainable innovation performance in manufacturing organizations. Procedia Cirp. 2016;40:449–54.

[24] Elkington J, Henriques A, Richardson J. The Triple Bottom Line: does it all add up. Adressing the Sustainability of Business and CSR New York City: Earthscan. 2004.

[25] Obamen J, Solomon O, Gabriel OO, Eluka J. Environmental management practices and sustainability of multinational companies in South-South, Nigeria. Journal of Business and Retail Management Research. 2019;13(3).

[26] Cao Y, Zhang Z, Guo Z, editors. Multi-objective flexible job shop scheduling problem with triple bottom line theory-based sustainable objectives. Journal of Physics: Conference Series; 2020: IOP Publishing.

[27] Yue G, Wei H, Khan NU, Saufi RA, Yaziz MFA, Bazkiaei HA. Does the environmental management system predict tbl performance of manufacturers? The role of green HRM practices and OCBE as serial mediators. Sustainability. 2023;15(3):2436.

[28] Hilman I, Djamil M, Saluy AB, Nurhayati M. Analysis of Entrepreneurship Sustainability in Individual SMEs (One-Man-Show) from A Triple Bottom Line (TBL) Perspective: Study in Depok City, Indonesia. Management Analysis Journal. 2023;12(1):137–43.

[29] Pan X, Sinha P, Chen X. Corporate social responsibility and eco‐innovation: The triple bottom line perspective. Corporate Social Responsibility and Environmental Management. 2021;28(1):214–28.

[30] Bassetti T, Blasi S, Sedita SR. The management of sustainable development: A longitudinal analysis of the effects of environmental performance on economic performance. Business Strategy and the Environment. 2021;30(1):21–37.

[31] Khan SAR, Zhang Y, Kumar A, Zavadskas E, Streimikiene D. Measuring the impact of renewable energy, public health expenditure, logistics, and environmental performance on sustainable economic growth. Sustainable development. 2020;28(4):833–43.

[32] Kocmanová A, Dočekalová M. Construction of the economic indicators of performance in relation to environmental, social and corporate governance (ESG) factors. Acta Universitatis Agriculturae et Silviculturae Mendelianae Brunensis. 2012;60(4):195–206.

[33] Cek K, Eyupoglu S. Does environmental, social and governance performance influence economic performance? Journal of Business Economics and Management. 2020;21(4):1165–84.

[34] Tariq A, Badir Y, Chonglerttham S. Green innovation and performance: moderation analyses from Thailand. European Journal of Innovation Management. 2019;22(3):446–67.

[35] Peyravi B, Jakubavičius A. Drivers in the eco-innovation road to the circular economy: Organiational capabilities and exploitative strategies. Sustainability. 2022;14(17):10748.

[36] Abbas J, Khan SM. Green knowledge management and organizational green culture: an interaction for organizational green innovation and green performance. Journal of Knowledge Management. 2023;27(7):1852–70.

[37] Schiederig T, Tietze F, Herstatt C. Green innovation in technology and innovation management–an exploratory literature review. R&d Management. 2012;42(2):180–92.

[38] Sun Y, Gao P, Tian W, Guan W. Green innovation for resource efficiency and sustainability: Empirical analysis and policy. Resources Policy. 2023;81:103369.

[39] Yurdakul M, Kazan H. Effects of eco-innovation on economic and environmental performance: Evidence from Turkey’s manufacturing companies. Sustainability. 2020;12(8):3167.

[40] Zandi G, Khalid N, Islam DMZ. Nexus of knowledge transfer, green innovation and environmental performance: impact of environmental management accounting. International Journal of Energy Economics and Policy. 2019;9(5):387–93.

[41] Ali S, Dogan E, Chen F, Khan Z. International trade and environmental performance in top ten‐emitters countries: the role of eco‐innovation and renewable energy consumption. Sustainable Development. 2021;29(2):378–87.

[42] Ch’ng P-C, Cheah J, Amran A. Eco-innovation practices and sustainable business performance: The moderating effect of market turbulence in the Malaysian technology industry. Journal of Cleaner Production. 2021;283:124556.

[43] Demirel P, Danisman GO. Eco‐innovation and firm growth in the circular economy: Evidence from European small‐and medium‐sized enterprises. Business Strategy and the Environment. 2019;28(8):1608–18.

[44] Munodawafa RT, Johl SK. A systematic review of eco-innovation and performance from the resource-based and stakeholder perspectives. Sustainability. 2019;11(21):6067.

[45] Byrne BM. Structural Equation Modeling With AMOS—Basic Concepts, Applications, and Programming. New York & London: Routledge; 2016. 460 p.

[46] We Shiyab, Ferguson C, Rolls K, Halcomb E. Solutions to address low response rates in online surveys. European journal of cardiovascular nursing. 2023;22(4):441–4. doi: 10.1093/eurjcn/zvad030 36827086

[47] Hair JF, Black WC, Babin BJ. Multivariate Data Analysis: A Global Perspective: Pearson Education; 2010.

[48] Xia Y, Yang Y. RMSEA, CFI, and TLI in structural equation modeling with ordered categorical data: The story they tell depends on the estimation methods. Behavior research methods. 2019;51(1):409–28. doi: 10.3758/s13428-018-1055-2 29869222

[49] Taber KS. The Use of Cronbach’s Alpha When Developing and Reporting Research Instruments in Science Education. Research in Science Education. 2017;48(6):1273–96. doi: 10.1007/s11165-016-9602-2

[50] Wheaton B, Muthen B, Alwin DF, Summers GF. Assessing Reliability and Stability in Panel Models. Sociological Methodology. 1977;8:84–136. doi: 10.2307/270754

[51] Carmines EG, McIver JP. Analyzing models with unobserved variables: Analysis of Covariance structures. G. W. Bohrnstedt EFBE, editor. Hills Beverly: Sage Publications Inc.; 1981 1981. 65–115 p.

[52] Bentler PM. Comparative fit indexes in structural models. Psychological Bulletin. 1990;107(2):238–46. doi: 10.1037/0033-2909.107.2.238 2320703

[53] Jöreskog KG, Sörbom D. Recent developments in structural equation modeling. Journal of Marketing Research. 1982;19(4):404–16. doi: 10.2307/3151714

[54] Marsh HW, Hau K-T, Wen Z. In Search of Golden Rules: Comment on Hypothesis-Testing Approaches to Setting Cutoff Values for Fit Indexes and Dangers in Overgeneralizing Hu and Bentler’s (1999) Findings. Structural Equation Modeling: A Multidisciplinary Journal. 2004;11(3):320–41. doi: 10.1207/s15328007sem1103_2

[55] Schaltegger S, Wagner M. Sustainable entrepreneurship and sustainability innovation: categories and interactions. Business strategy and the environment. 2011;20(4):222–37.

[56] Delmas MA, Pekovic S. Environmental standards and labor productivity: Understanding the mechanisms that sustain sustainability. Journal of Organizational Behavior. 2013;34(2):230–52.

[57] Horbach J, Rammer C. Energy transition in Germany and regional spill-overs: The diffusion of renewable energy in firms. Energy policy. 2018;121:404–14.

[58] Horbach J, Rammer C, Rennings K. Determinants of eco-innovations by type of environmental impact—The role of regulatory push/pull, technology push and market pull. Ecological economics. 2012;78:112–22.

[59] Tukker A. Product services for a resource-efficient and circular economy–a review. Journal of cleaner production. 2015;97:76–91.

[60] Bossle MB, de Barcellos MD, Vieira LM, Sauvée L. The drivers for adoption of eco-innovation. Journal of Cleaner production. 2016;113:861–72.

[61] Delmas MA, Pekovic S. Corporate sustainable innovation and employee behavior. Journal of business ethics. 2018;150:1071–88.

[62] Muller A, Kolk A. Extrinsic and intrinsic drivers of corporate social performance: Evidence from foreign and domestic firms in Mexico. Journal of Management studies. 2010;47(1):1–26.

[63] Reyes-Rodríguez JF, Contreras-Pacheco OE, Arias OPC. Sustainability-oriented innovations and value creation in SMEs: An illustration in the Colombian context. Management. 2023;21(3):777–91.

